# Interplay of Phosphorylated Apoptosis Repressor with CARD, Casein Kinase-2 and Reactive Oxygen Species in Regulating Endothelin-1–Induced Cardiomyocyte Hypertrophy

**Published:** 2013-08

**Authors:** Iram Murtaza, Hong-Xia Wang, Sobia Mushtaq, Qamar Javed, Pei-Feng Li

**Affiliations:** 1 Division of Cardiovascular Research, National Key Laboratory of Biomembrane and Membrane Biotechnology, Institute of Zoology, Chinese Academy of Sciences, Beijing 100080, People's Republic of China; 2 Department of Biochemistry, Faculty of Biological Sciences, Quaid-i-Azam University Islamabad, 45320, Islamabad, Pakistan

**Keywords:** Apoptosis repressor with caspase recruitment dom- ain, Cardimyocyte hypertro- phy, Endothelin-1, Protein kinase CK2, Reactive oxygen species

## Abstract

***Objective(s):*** The role of the Apoptosis repressor with caspase recruitment domain (ARC) in apoptosis and in certain hypertrophic responses has been previously investigated, but its regulation of Endothelin-1 induced cardiac hypertrophy remains unknown. The present study discusses the inhibitory role of ARC against endothelin–induced hypertrophy.

***Results:*** In present study Endothelin treated cardiomyocytes were used as a hypertrophic model, that were subsequently treated with adenovirus ARC and its mutant at different multiplicity of infections. Casein-kinase-2 inhibitors were used to produce dephosphorylated ARC and to study its effect on hypertrophy. Hypertrophy was assessed by cell surface area measurement, Atrial-natriuretic-Factor mRNA analysis and total protein assay. Reactive oxygen species analysis was carried out using the dichlorofluorescin-diacetate (DCFH-DA) assay. Over expression of ARC significantly inhibits Endothelin–induced cardiomyocyte hypertrophy. The nonphosphorylated mutant ARC (T149 A) remained unable to control endothelin–induced hypertrophy, suggesting a vital role for ARC phosphorylation in regulation of its activity. Sensitization study has been carried out to check the role of endogenous ARC using casein-kinase inhibitors. Finally, the significant role of ARC in regulating reactive oxygen species -mediated control of endothelin induced hypertrophy has also been assessed.

***Conclusion*** *:* Conclusively, present study showed the vital and potential therapeutic interventional role of ARC in preventing endothelin-1–induced cardiomyocyte hypertrophy. The regulation of hypertrophic pathway by ARC relies on blunting the reactive oxygen species attack. This study further suggests a mediatory role of casein-kinase-2 in Endothelin–induced hypertrophy, mainly through its phosphorylation of ARC.

## Introduction

Sustained cardiac hypertrophy is often accompanied by maladaptive cardiac remodeling, leading to heart failure (1). A fundamental insight into the biology of cardiac hypertrophy is vital to the continuing battle against this common and deadly disease (2). Signaling pathways that mediate cardiac hypertrophy have been investigated for many years; however, the nature of the relationships between these pathways remains to be elucidated.

The apoptosis repressor with caspase-recruitment domain (ARC) is abundantly expressed in the heart, which makes it a unique and central cardioprotective agent for the heart (3). Many studies have explored its role as an antiapoptotic factor (3, 4). Hypertrophy and apoptosis are two distinct cellular events, but both have several stimuli in common. Previous studies have shown that angiotensin II (Ang II) and tumor necrosis factor- (TNF-) can induce both hypertrophy and apoptosis (5). Furthermore, apoptosis may drive compensated hypertrophy to failure in the work-overloaded myocardium (6). In a previous study by the current authors, they have successfully elucidated the role of ARC in preventing phenylephrine (PE)-, TNF--, and Ang II–induced cardiac hypertrophy (1). However, the role of ARC in endothelin 1 (ET-1)–induced hypertrophy remain enigmatic, which is addressed in the present study.

Prolonged exposure of cardiomyocytes to external stimuli, hemodynamic overload, and neurohormonal factors such as ET-1 lead to pathological cardiac hypertrophy (7). ET-1 is a vasoactive peptide that contains 21 amino acids and has 2 intramolecular disulfide bonds (8). The endothelin peptide is expressed in a variety of cells, as cardiac smooth muscle cells and bronchial smooth muscle cells and can lead to cellular remodeling (9, 10), and it has potent mitogenic and vasoconstrictive effects (11). *In vitro* studies in the neonatal rat have shown that ET 1–induced cardiac hypertrophy involves various hypertrophic signaling cascades, such as those involving protein kinase, Raf-1, and mitogen-activated protein kinases, which are mediated by the ET–type A (ETA) receptors (12). Regarding the role of ET-1 *in vivo*, it is found to be markedly increased in the hypertrophied heart and the failing heart, conditions that are, interestingly, significantly inhibited by chronic treatment with ETA-receptor antagonists (13). In total, these data confirm a significant role for ET-1 in the development of cardiac hypertrophy *in vitro* and *in vivo*. Thus far, the effects of ET-1 on cardiac hypertrophy have been well documented; nevertheless, little is known about the possible therapeutic interventions and their underlying signaling pathways.

Reactive oxygen species (ROS)-generating pathway is one among the complex signal-transduction pathways that can mediate hypertrophic signals. ROS can mediate the hypertrophic signals of TNF-α, PE, Ang II (1), and ET-1 (14). Protein kinase CK2 (CK2) is a serine/threonine protein kinase, and its expression is ubiquitous in eukaryotic cells. It plays a key role in control of the cell cycle and cellular differentiation-and-proliferation. CK2 is characterized by its constitutive activation, and it phosphorylates ARC at T149 (15). Recent studies showed that ARC has the ability to inhibit different apoptotic pathways by blocking FAS-FADD binding and assembly of death –induced signaling complex. This is achieved by reducing the activity of caspase 2 and 8 and by blocking BOX activation (16). ARC has the ability to inhibit mitochondrial fission by binding PUMA that inhibit Drp1 accumulation in mitochondria, by blocking Smac/DIABLO release and thus maintain mitochondrial membrane potential (17). The present study confirms that the constitutively expressing phosphorylated ARC can prevent ET 1–induced hypertrophy. The antihypertrophic effect of ARC occurs through the scavenging of ROS generated due to ET-1 stimuli. Furthermore, the current study also shows the augmenting role of CK2, which is believed to be responsible for ARC phosphorylation at the endogenous level, in inhibiting ET1–induced hypertrophy. 

## Materials and Methods

Animal studies were performed in compliance with animal-welfare regulations of local authorities. ET-1, TBB, DRB, and Phalloidin tetramethylrhodamine isothiocyanate were purchased from Sigma (St. Louis, MO). 2’, 7’-dichlorofluorescin diacetate (DCFH-DA), was purchased from Molecular Probes Inc.

***Construction of adenoviruses harboring ARC***

The adenoviruses harboring the wild-type rat ARC (AdARC) and an ARC mutant with T149 converted to the alanine residue (AdT149Aββ^1^).

***Preparations and transfection of ARC antisense oligonucleotides***

-ARC antisense oligonucleotides were synthesized to inhibit endogenous ARC expression. The sequences of phosphothioate-modified antisense oligonucleotides targeted to ARC were ARC antisense oligonucleotides (ARC-AS), 5’-TGGGCATGGAGGGTCATAGCT-3’; scrambled ARC antisense oligonucleotides (S-ARC-AS), 5’-GTAGGCTGAGGTCGATCGGTA-3’ and ARC sense oligonucleotides (ARC-S), 5’-AGCTATGACCCTCCATGCCCA-3’. The specificity of the oligonucleotides was confirmed by comparison with all other sequences in Genbank using Nucleotide BLAST. There was no homology to other known rat DNA sequences. Cells were transfected with the oligonucleotides by using lipofectin (Life Technology). 

***Isolation and culture of cardiomyocytes***

Cardiomyocytes were isolated from 1- to 2–day-old Wistar rats as previously described (18, 19). Experiments were carried out strictly according to the guidelines issued by the *National Institutes of Health* (NIH, USA). Briefly, hearts were washed after dissection, minced in N-2-hydroxyethylpiperazine-N'-2-ethanesulfonic acid –buffered saline solution containing (in mM): NaCl, KCl, NaH_2_PO_4_, glucose, and HEPES in the ratio 130:3:1:4:20 (pH adjusted to 7.35 with NaOH). The tissues were then dispersed in a series of incubations at 37°C in HEPES-buffered saline solution containing 1.2 mg/ml pancreatin and 0.14 mg/ml collagenase (Worthington). After centrifugation, the cells were resuspended in Dulbecco’s modified Eagle’s medium/F-12 (GIBCO) containing 5% heat-inactivated horse serum, 0.1 mM ascorbate, insulin-transferring-sodium selenite media supplement, 100 U/ml penicillin, 100 µg/ml streptomycin, and 0.1 mM bromodeoxyuridine. The dissociated cells were preplated at 37°C for 1 hr. The cells were then diluted to 1 10^6^ cells/ml and plated in different culture dishes coated with 10 µg/ml laminin, according to specific experimental requirements. After 24 hr, the medium was replaced by a serum-free medium.

***Adenovirus infection and CK2 inhibition***

Cardiomyocytes were infected with adenoviruses as previously described (1). In experiments applying the CK2 inhibitor, cells were pretreated with TBB and DRB for 50 min and 24 hr, respectively, before inducing hypertrophy.

***Cell surface–area measurement***

Cell-surface areas of F-actin stained cells or unstained cells were measured after applying hypertrophic stimuli by computer-assisted planimetry. To determine the changes in cell size, the peripheries of cell images captured by a charge-coupled device camera (Olympus, Tokyo, Japan) were traced and analyzed using NIH Image software. In each experiment, 100–200 cardiomyocytes were examined in 20–50 fields.

***(*** ^3^ ***H) leucine incorporation***

Cardiomyocytes were infected with AdARC or Adβ-gal. 24 hr after infection they were treated with the hypertrophic stimuli for 48 hr in the presence of (^3^H) leucine (1.0 µCi/ml). Thereafter, cells were washed 3 times with PBS, incubated with 5% trichloroacetic acid for 20 min at 4°C, and lysed with 0.5 M NaOH. Scintillation fluid was applied to the lysates, and the mixtures were counted in a liquid scintillation counter.

***Analysis of ANF expression by Northern blotting***

Atrial natriuretic factor (ANF) expression was detected by Northern blotting as reported previously (1). Briefly, pre-hybridization was conducted at 42°C for 4 hr in a pre-hybridization buffer: 50% formamide, 5x SSC, 2% blocking reagent, 50 mM sodium phosphate, pH 7.4, 7% SDS (wt/vol), and 0.1% *N*-laurylsarkosine (wt/vol). Hybridization was performed in the same buffer and temperature for 30 hr with digoxigenin-labeled ANF cDNA probe. For chemiluminescent detection, the membrane was blocked for 30 min in 2.5% blocking reagent and then incubated for 30 min with anti-digoxigenin antibody conjugated with alkaline phosphatase. After two washes with 100 mM maleic acid buffer containing 0.3% Tween-20, CSPD substrate solution was added to the membrane and incubated for 10 min. The same membrane was stripped and re-probed with glyceraldehyde-3-phosphate dehydrogenase (GAPDH) as a loading control.

***Immunoblotting***

Immunoblotting was performed as described (15). In brief, cells were lysed for 1 hr at 4°C in a lysis buffer [in mM] 20 Tris [pH 7.5], 2 EDTA, 3 EGTA, 2 DTT, 250 sucrose, 0.1 PMSF, 1% Triton X-100 and a protease inhibitor cocktail). Samples were subjected to 12% SDS-PAGE and transferred to nitrocellulose membranes. Equal-protein loading was controlled by Ponceau red staining of membranes. Blots were probed using antibodies.

***Intracellular ROS analysis***

Intracellular ROS levels were analyzed using the ROS-sensitive dye, DCFH-DA, as described (1). DCFH-DA was employed to measure ROS. DCFH-DA dissolved in absolute ethanol (20 mM), was used at a final concentration of 20 µM. Hydrogen peroxide is able to oxidize DCFH to the fluorescent DCF. Cells were harvested and suspended in DMEM medium with 0.2% fetal calf serum. ROS probes were then added and incubated for 30 min at 37°C. 

Hydrogen peroxide (H_2_O_2_; 30% w/v) was diluted in distilled water to a 20 mM stock solution and used at a final concentration of 200 µM as a positive control because of its known capacity to induce intracellular oxygen radical and hydrogen peroxide production in human cells. The fluorescence of 2',7'-dichlorofluorescein (DCF) was measured by flow cytometry and confocal fluorescence intensity–imaging microscope.

***Statistical analysis***

The results are expressed as mean values SEM. The statistical comparison among different groups was carried out by one-way ANOVA. Paired data were evaluated by Student's t-test. A level of p <0.05 was considered statistically significant.

## Results

***ARC is able to inhibit ET 1–induced cardiomyocyte hypertrophy***

**Figure 1:**
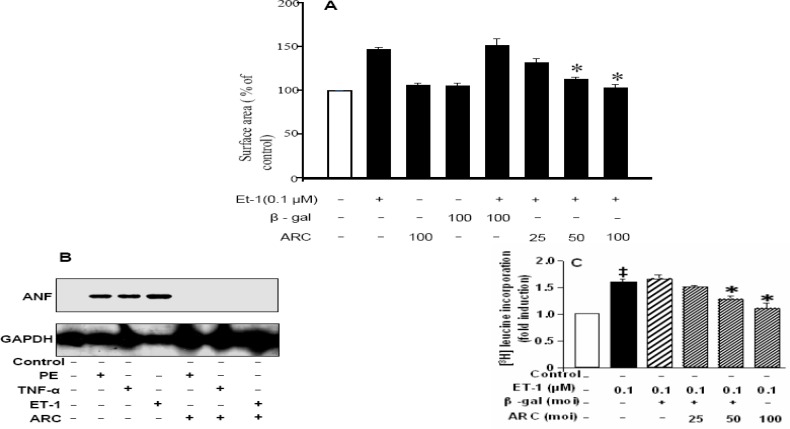
ARC inhibits ET 1–induced hypertrophic responses. The cultured neonatal rat cardiomyocytes were infected with adenovirus ARC (AdARC) and viral control, adenovirus-galactosidase construct (Ad -gal) at varied multiplicity of infection (moi). 24 hr after infection, they were treated with hypertrophic stimuli of 0.1μmol/L ET-1 for 24 hr. A): The cell surface area was measured by the computer-assisted planimetry of 200 cells in 40 to 50 fields. B): Total protein content was analyzed through [^3^H] Leucine incorporation method C): Hypertrophic marker ANF RNA level was assessed by northern blotting. In addition to Et-1 other hypertrophic inducers as PE, and TNF were also tested for comparison. The data are indicated as mean ± SEM of 3 independent experiments. *P <0.05, vs ET-1 alone and ET-1 in the presence of viral control

To delineate the inhibitory role of ARC in neurohormone-induced cardiomyocyte hypertrophy, it was examined whether phosphorylated ARC can block this route of hypertrophic induction. Wild-type phosphorylated ARC adenovirus (AdARC) was expressed at a multiplicity of infection 100, whereas Ad-gal was considered the adenoviral control. Appropriate multiplicities of infection of adenoviruses were determined after numerous experiments with varying ranges. The cardiomyocyte hypertrophic model was set up by applying 0.1 µM ET-1 as described (20, 21). As sarcomeric organization and increase in myocyte perimeter (22) is major marker of cardiomyocyte hypertrophy, the cell-surface area was measured. Cell-surface area data showed that the significant increase in surface area after treatment with ET-1 was blocked by treatment with wild-type phosphorylated ARC (Figure 1 A). To confirm the role of ARC at molecular level in hypertrophy, Atrial natriuretic factor (ANF) RNA expression after ET-1 treatment was significantly reduced (Figure 1 B, last lane) like the treatment with already known hypertrophic stimuli as TNF and PE (Figure 1 B). Further during ET-1 induced maladaptive cardiac hypertrophy, total protein level of cardiomyocytes is significantly increased as analyzed through (^3^H) leucine incorporation method. This increase can be prevented by ARC overexpression (Figure 1C). These results concluded that ARC overexpression acts at molecular level of hypertrophic pathway and plays a dynamic role to antagonize ET-1–induced cardiomyocyte hypertrophy.

***ARC mutant, nonphosphorylated at 149 position, unable to inhibit ET 1–induced cardiomyocyte hypertrophy***

**Figure 2:**
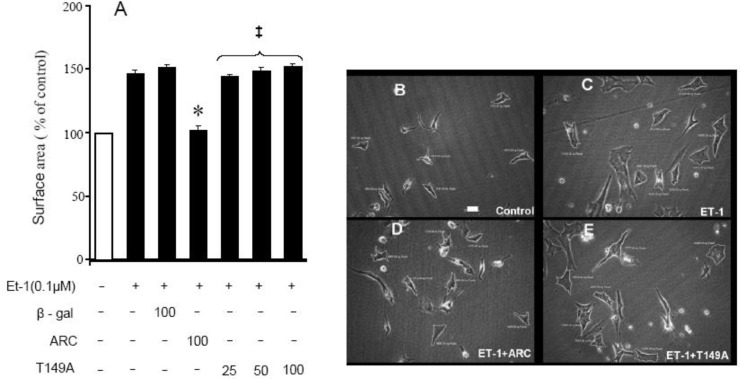
The mutant ARC, unphosphorylated at the 149th position, unable to inhibit the ET 1–induced cardiomyocyte hypertrophy and endogenous sensitization with ARC by applying its sense strand. A: Cultured neonatal rat cardiomyocytes were infected for 24 hr with different “moi” of adenovirus ARC (ARC), nonphosphorylated mutant (T149A), or viral control (Adβ-gal). After infection, they were stimulated for 24 hr with 0.1 μM ET-1. The cell-surface area was analyzed by measuring 200 cells in 40 to 50 fields. The data indicated are mean ± SEM of 3 independent experiments. *P < 0.05, vs ET-1 alone and ET-1 in presence of viral control, ‡*P >* 0.05, vs ET-1 alone and ET-1 in presence of viral control. Photographs of cultured neonatal rat cardiomyocytes were obtained at 100x resolution, bar = 600 pixels; B: control; C: 24 hr after applying ET 1–induced hypertrophic stimuli; D: CMC treatment with 100 moi AdARC, followed by 24 hr ET-1 stimuli; E: CMC treatment with nonphosphorylated ARC mutant T149 A, followed by ET-1 stimuli

βFigure 2 A). The present data confirmed the 149th position of ARC as being a crucial functional phosphorylated site that is vital for ARC inhibition of ET 1–induced cardiomyocyte hypertrophy (Figure 2 B–E). 

**Figure 3:**
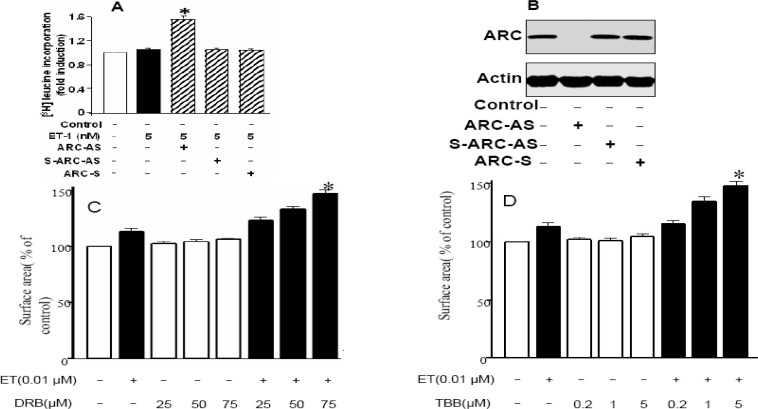
Inhibition of endogenous ARC phosphorylation sensitizes cardiomyocytes to undergo ET-1 induced hypertrophy. *A*: ARC antisense (ARC-AS), scrambled ARC antisense (S-ARC-AS) and ARC sense strands (ARC-S) treated cardiomyocytes were stimulated with Et-1 (5nM). Fold increase in total protein level was analyzed by [^3^H] Leucine incorporation method. *B*: Western Blot confirming ARC anti sense (ARC-AS) strand inhibited endogenous ARC production, Lane 2. The cultured neonatal rat cardiomyocytes were incubated with different doses of CK2 inhibitors. *C:* treatment with DRB to check its dose-dependent effect; 24 hr after incubation with different doses of DRB (25, 50, and 75μM), cells were stimulated with 0.01 μM ET-1. Cell-surface area was measured and data are expressed as the mean ± SEM of 3 independent experiments; *P < 0.05, vs 0.01 μM ET-1. *D:* TBB group–0.2, 1, and 5 M TBB (50 min incubation)–treated group; *P < 0.05, vs ET-1. The data indicate mean ± SEM of 3 independent experiments

***Inhibition of Endogenous ARC phosphorylation sensitizes cardiomyocytes to undergo ET 1–induced hypertrophy***

In this phase of ARC sensitization experiments, endogenous ARC role in cardiomyocytes hypertrophy was analyzed by applying ARC antisense strand. Here very low dose of ET (5 nM) was applied that have no effect on cardiomyocytes hypertrophy as assessed by (3H) leucine incorporation method, but ARC antisense strand treatment inhibited endogenous ARC and sensitized cardiomyocytes to undergo hypertrophy (Figure 3 A). ARC antisense strand inhibition of endogenous ARC was confirmed through western blot in Figure 3 B. 

For a better understanding of dependence of ARC on phosphorylation for its antihypertrophic effect, the authors carried out a study with the dephosphorylation of endogenous ARC. Because physiologically ARC is constitutively phosphorylated by CK2 (15), CK2 inhibitors DRB and TBB were used (23μFigure 3 C-D). These results clearly depicted the physiologically important role of CK2 in phosphorylating ARC and its subsequent involvement in inhibition of ET 1–induced hypertrophy.

***ARC can control ET 1–induced cardiomyocyte hypertrophy by controlling intracellular ROS***

To confirm the hypertrophic pathway followed by ET-1 and its subsequent inhibition by ARC, experiments to check the prevention of ET 1–induced increase in ROS levels by ARC were carried out. This study is also supported by the previous work by the authors of this study depicting regulation of catalase activity by ARC (1). 

**Figure 4:**
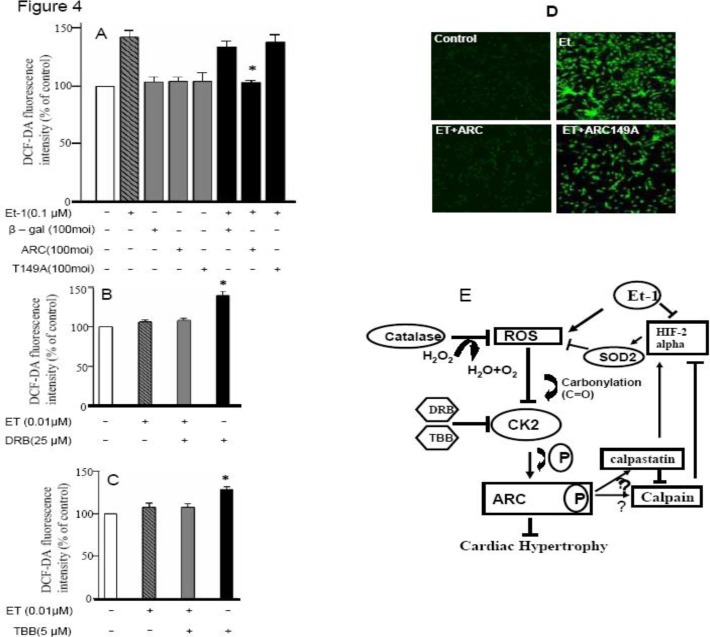
ARC can control ET 1–induced cardiomyocyte hypertrophy by controlling intracellular ROS. A: The cultured neonatal rat cardiomyocytes were infected with adenovirus ARC (AdARC), nonphosphorylated ARC mutant (AdT149 A), or adenovirus-galactosidase construct (Adβ-gal) at the indicated multiplicity of infection (100 moi); 24 hr after infection, they were incubated with 5 μM DCFDA for 30 min at 37^o^C in the presence of 0.1 μM ET-1. Data are expressed as the mean ± SEM of 3 independent experiments. *P <0.05 vs ET-1 + Adβ-gal. B: The cultured neonatal rat cardiomyocytes were incubated with 25 μmol/L DRB; 24 hr after incubation, they were incubated with 5 μM DCFH-DA for 30 min at 37 ^o^C in the presence of 0.01 μM ET-1. Data are expressed as the mean   SEM of 3 independent experiments, *P <0.05 vs ET-1. C: 5 μM TBB (50 min incubation)–treated group, *P <0.05 vs ET-1. The data indicate mean ± SEM of 3 independent experiments. D: Representative photographs of control and treated cardiomyocytes from confocal microscope showing fluorescence intensity. E: Hypothetical pathway depicting different events involved during the ARC regulation of ET 1–induced cardiomyocyte hypertrophy

Cardiomyocytes were treated with ARC and its nonphosphorylated mutant after hypertrophic stimulation with ET-1. Reactive oxygen species were detected by dichlorodihydrofluorescein diacetate fluorescence-intensity measurements. These results significantly showed the control of ET 1–induced ROS levels by ARC, whereas its mutant was unable to blunt the increased levels of ROS (Figure 4 A). The authors also studied whether endogenous ARC depends on phosphorylation for the control of hypertrophy by blunting of the ROS pathway. With this objective, the authors used CK2 inhibitors with low doses of ET-1 and estimated the ROS levels both with and without ARC treatment (Figure 4-B, C). Representative confocal images for ROS intensity clearly showed ARC anti ET-1 induced hypertrophy role (Figure 4-D). These results indicate that inhibition of endogenous ARC phosphorylation leads to the increased susceptibility of cells to ET 1–induced hypertrophy under ROS activation. We also hypothesized a pathway that showed through ARC, CK-2 and ROS interplay regulation of neurohormone (ET-1) induced cardiac hypertrophy (Figure 4 E). 

## Discussion

The findings of the present study are important because they show for the first time that ARC treatment can prevent the neurohormone ET-1–induced cardiomyocyte hypertrophy *in vitro* by blunting the effects of ROS. Previous studies have indicated that ET-1 endogenous levels are also increased in the case of Ang II–induced cardiomyocyte hypertrophy (24). These increased ET-1 levels are responsible for the progression of hypertrophy. Previous studies have confirmed that ET-1 can increase the expression of caspase-8 (25). Caspase-8 is responsible for the release of ROS and cytochrome-c from the mitochondria and can cause severe physiological disorders including apoptosis. ARC can also directly bind to caspase-8 through its CARD domain and plays a vital role in inhibiting apoptosis induced by a variety of stimuli requiring the engagement of these caspases (3). These studies strongly support the data obtained in this study and provide a clue for the protective role of ARC in ET 1–induced cardiomyocyte hypertrophy. 

It has been reported that the 5′-flanking region of the ppET-1 gene contains the TPA-responsive elements (TRE). These responsive elements provide the binding site to the gene products c-fos and c-jun responsible for hypertrophy and apoptotis (26). 

ROS, an important mediator of hypertrophy, plays an important role in neurohormone-induced hypertrophy because it has been shown to regulate the endogenous level of c-fos through the adapter protein 1 (AP-1) or Ras pathway under ET-1 stimulation (27). Furthermore, ET-1 can lead to PKC activation (28), which can generate ROS in a nicotinamide adenine dinucleotide phosphate oxidase (NADPH oxidase)–dependent manner (29). 

The current studies indicate clearly that ARC can abrogate the ET 1–induced hypertrophy, whereas endogenous ET-1 can lead further to hypertrophy if inhibition of CK-2 occurred. In present study we have used varied concentrations of ET-1 under different scenarios. In order to induce hypertrophy ET 0.1 µM (Figure 1 and 2) was used but in sensitizing experiments such doses of ET were selected that by themselves were unable to induce hypertrophy as 0.01 µM and 5nM. These negligible doses of ET in combination with CK-2 inhibitors showed hypertrophic responses (Figure 3). 

Previous reports mentioned that ET-1 stimulation contributes to different cardiac disorders by activating the vascular endothelial growth factor (VEGF), Ap-1, Jun N-terminal kinase–, extracellular signal-regulated kinase–, and ROS-related pathways (9, 21). ET-1 downregulation by carnitine can control ischemic cardiovascular diseases by mitigating noxious effect of free redicals in reperfused hearts (30). ET-1 can activate the hypoxia-inducible factor 1-alpha (HIF-1-alpha) by reinduction of calcium (31) and downregulates the HIF-2 alpha. HIF-1 alpha has been shown to be activated under both hypoxic conditions and under active NADPH-oxidase conditions (32). This can further lead to increased AP 1–mediated activation of VEGF and cardiac hypertrophy. HIF-2 alpha is reported to be downregulated at the molecular level under hypertrophic conditions (33); furthermore, its intracellular activity is regulated by calpain under stress conditions. The endogenous inhibitor of calpain, calpastatin, can restore the levels of HIF-2 alpha and its resultant superoxide dismutase (SOD2) activity (34). SOD may further link to or share its function with catalase to modulate the activity of CK2 by preventing its ROS–mediated corbonylation. 

Previous studies have reported the subcellular localization of different isoforms of caplain in the cytosol (μ and m) and in the mitochondria (I and II) (31). It was proved that it may be localized in the mitochondrial matrix or in the mitochondrial membrane (35). Calpain inhibition under normal physiological conditions can be achieved by its endogenous inhibitor calpastatin. A few studies have shown that calpastatin is only localized in the cytosol (36). Phosphorylated ARC is abundantly localized in the mitochondria. The previous work of the authors of this study also supports its protective functioning through its mitochondrial localization (1). Furthermore, the current study suggested strongly that continuous phosphorylation of ARC by CK-2 causes phosphorylated ARC to function inside the mitochondria; moreover, it also showed that the ET 1–induced increase in ROS is blunted by ARC and can depict the strong relation of ARC with calpain regulation inside the mitochondria. Several studies also reported calpastatin localization and its inhibition of μ-caplain inside the mitochondria (35). In this study we just hypothesize that ARC may influence the upregulation of calpastatin or regulate its maintenance inside mitochondria by maintaining either a normalized mitochondrial permeability transition or help to maintain localization of calpastatin in the mitochondria to control the action of membrane-bound calpain. ARC has been reported to be a potent protective agent against hypoxia induced pulmonary arterial smooth muscle cell death and hypoxia-induced downregulation of selective voltage-gated potassium channels (37). It is recently reported that calpain deficiency lead to mitochondrial dysfunction, fission and mitophagy too (38).

The current study shows that ARC can block the cascade of hypertrophic stimuli by blunting the ROS pathway. Furthermore, it can be hypothesized from the current study that there may be a direct inhibitory relationship between the (i) ROS-activated AP-1, cfos, VEGF, HIF-1α, and ARC-related control of HIF-2 in the mitochondria and (ii) the presentation of SOD and catalase for reinduction of CK-2 activity (Figure 4D).

## Conclusion

The antihypertrophic effect of ARC occurs through the scavenging of ROS generated because of neurohormone, ET-1 stimuli. Furthermore, the current study also shows the augmenting role of CK2, which is believed to be responsible for ARC phosphorylation at the endogenous level, in inhibiting ET1–induced hypertrophy. Future *in vivo* research in the mouse model, based on the findings of current studies and presented hypothesis, can lead to novel therapeutic approaches for the treatment of maladaptive cardiac hypertrophy leading to cardiac failure. 
